# Childbirth experience, risk of PTSD and obstetric and neonatal outcomes according to antenatal classes attendance

**DOI:** 10.1038/s41598-022-14508-z

**Published:** 2022-06-23

**Authors:** Valérie Avignon, David Baud, Laurent Gaucher, Corinne Dupont, Antje Horsch

**Affiliations:** 1 Department Woman-Mother-Child, Lausanne University Hospital (CHUV), Avenue Pierre-Decker 2, 1011 Lausanne, Switzerland; 2 Research On Healthcare Performance RESHAPE, INSERM U1290, Université Claude Bernard Lyon 1, Domaine Rockefeller 8 avenue Rockefeller 69373 Lyon cedex 08, Lyon, France; 3 Institute of Higher Education and Research in Healthcare (IUFRS), University of Lausanne, Route de la Corniche 10, 1010 Lausanne, Switzerland; 4 Geneva School of Health Sciences, HES-SO University of Applied Sciences and Arts, Western Switzerland, Geneva, Switzerland

**Keywords:** Psychology, Health care, Patient education

## Abstract

Antenatal classes have evolved considerably and include now a discussion of the parents' birth plan. Respecting this plan normally results in a better childbirth experience, an important protective factor of post-traumatic stress disorder following childbirth (PTSD-FC). Antenatal class attendance may thus be associated with lower PTSD-FC rates. This cross-sectional study took place at a Swiss university hospital. All primiparous women who gave birth to singletons from 2018 to 2020 were invited to answer self-reported questionnaires. Data for childbirth experience, symptoms of PTSD-FC, neonatal, and obstetrical outcomes were compared between women who attended (AC) or not (NAC) antenatal classes. A total of 794/2876 (27.6%) women completed the online questionnaire. Antenatal class attendance was associated with a poorer childbirth experience (*p* = 0.03). When taking into account other significant predictors of childbirth experience, only induction of labor, use of forceps, emergency caesarean, and civil status remained in the final model of regression. Intrusion symptoms were more frequent in NAC group (M = 1.63 versus M = 1.11, *p* = 0.02). Antenatal class attendance, forceps, emergency caesarean, and hospitalisation in NICU remained significant predictors of intrusions for PTSD-FC. Use of epidural, obstetrical, and neonatal outcomes were similar for AC and NAC.

## Introduction

Over the last decades, many changes have occurred in perinatal care. The arrival of the epidural in obstetrics in 1972 and its increasing access in the 1980s also profoundly modified the preparation for birth, previously mainly focused on pain management^1^. Antenatal classes have integrated a psychological preparation of the woman and her partner in addition to the physical preparation^2^, as psychological preparation may improve mental health during pregnancy^3^. Furthermore, couples are encouraged to participate actively in their birth project, starting during pregnancy^4^.

The "classical" preparation for birth is a legacy of the psycho-prophylaxis training introduced in France by Lamaze in the early 1950s. From 1955, this model of antenatal classes, also named "painless childbirth", was adopted in French-speaking Switzerland^5^. Partly because of the feminist movements and the decreasing length of stay in maternity wards, the future co-parent became an active participant of antenatal classes: he/she took on the role of supporter and coach during childbirth, as well as support for the return home after birth. The role of those in charge of birth preparation, often midwives, has also changed. Often employed by hospitals, their new teaching model is still based on informing future parents about pregnancy, childbirth, breastfeeding, and childcare. However, they must also promote the medical environment in which the birth will take place^1^.

In Switzerland, the “classical” birth preparation today is still based on the teaching of theoretical knowledge about pregnancy and childbirth, breathing techniques, and postural labor combined with relaxation^6–9^. However, these courses are now integrated in the birth plan that parents are invited to draw up, as recommended by the Haute Autorité de Santé (HAS)^10^, so that the couple's expectations can be taken into account. In addition, future parents are expected to develop specific skills, such as understanding and using information, and developing personal resources throughout the courses, thus marking the definitive shift from an objective of pain-free childbirth to one of psychological preparation for childbirth and parenthood. In the canton of Vaud, the usual costs of in-hospital sessions range from 160 to 344$, while the Swiss compulsory health insurance reimburses 160$ for an individual or group course given by a midwife^11^. These costs can represent a barrier to accessing antenatal classes.

Although many women and their partners have a positive childbirth experience, approximately one-third perceive their childbirth to be traumatic^12^. Between 3–6% of women in community samples (low risk) and between 6 and 18% of women in high-risk groups (e.g., preterm birth, emergency cesarean section) develop post-traumatic stress disorder following childbirth (PTSD-FC)^13,14^. PTSD-FC consists of four symptom clusters: re-experiencing of the traumatic event (intrusions), cognitive and behavioral avoidance, negative alterations in mood and cognitions, and hyperarousal^15,16^. A recent study showed that intrusion is the most common symptom after a childbirth-related trauma^17^. This may be due to the impact of pain, both in the bodily memory of the trauma but also as a reminder of the experience. The presence of the newborn near her mother can potentially also be a constant reminder of the traumatic experience, thus triggering intrusions^17^.

PTSD-FC can be influenced by a number of factors according to the diathesis–stress model of PTSD-FC^18^. Childbirth experience is one of these risk factors, as well as the mode of childbirth, the fear of childbirth, mental health status during pregnancy, and social support. The childbirth experience is a self-assessment of what a woman remembers about her birth^19^, sometimes even many years later^20,21^. More than just satisfaction with pain or care, the childbirth experience attempts to "measure feelings of control, expectancy satisfaction, confidence, and participation in decision making"^19^, p. 3. Questionnaires addressing the chilbirth experience assess both maternal satisfaction with the provided care and experience of birth^22^. The childbirth experience can therefore hardly be considered without the provided care. This is why some authors describe the childbirth experience as a subjective experience of birth, whereas the provision of care (mode of delivery, mode of anaesthesia/analgesia, duration of labor, state of the child at birth, etc.) is described as an objective experience^23^.

Recent studies have shown that antenatal classes could prevent PTSD-FC. A prospective study showed that participation in antenatal classes was a predictor of PTSD-FC symptoms at four months post-partum (t = − 2.15, β = − 0.15: *p* < 0.05) in a sample (n = 275) of nulliparous and multiparous women, independent of the content of these antenatal classes^24^. A randomised controlled trial highlighted that women who attended a specific program of antenatal classes had less PTSD-FC symptoms^25^. However, these results are difficult to generalise due to the large differences in content, teaching methods, and populations^26,27^. In this context, the aim of this study was to compare women who had participated in antenatal classes with those who had not regarding their childbirth experience, PTSD-FC, as well as their obstetric and neonatal outcomes.

## Methods

### Design

This cross sectional study took place at a Swiss university hospital.

### Setting

The Lausanne University Hospital has about 3200 births per year of all levels of complexity. The obstetrician following the pregnancy, especially for primiparous women, often recommends antenatal classes. Women have the option of enrolling in antenatal classes either at the hospital where they are due to give birth or with an independent midwife.

### Study procedure and participants

A Short Message Service (SMS) was sent to all primiparous women, 18 years or older, who gave birth to a single, alive, term baby (≥ 37 weeks of Gestational Age (GA) at the hospital between January 2018 to September 2020. This SMS invited them to participate in the study via an internet link after validation of an E-consent form. The SMS and the online questionnaires were available in French and English. Three reminders were sent from June 2020 to December 2020. Data and consent were collected and managed using REDCap electronic data capture tools^28^.

### Ethical issues

This project was developed in accordance with the rules and regulations in force in Switzerland. Informed consent was obtained from all participants as an E-consent form where women must complete their name, surname, birth date, date of completion and validate with a radio button their participation in the study on the RedCap system. The E-consent form was saved in the RedCap system and the participant had the option of downloading it with the answers of the information that she had completed. The Ethical Committee of the Canton de Vaud approved the study protocol (n°2019-02228).

### Measures

The primary outcome was the childbirth experience, as measured with the Childbirth Experience Questionnaire (CEQ-2)^29^. The English version of the questionnaire was translated into French using the forward–backward translation method and cultural adaptation proposed by Wild et al.^30^. In this study, the Cronbach alpha was calculated as 0.93. This 22-item questionnaire was designed to evaluate different aspects of primiparous' childbirth experiences and has four subscales: Own capacity (strengths, emotions, bodily sensations and its own ability to cope), Professional support (privacy, dignity, respect, the compassion and the understanding of the care team), Perceived safety (sense of security and the sensation of competency of the team perceived by the woman), and Participation (the woman receive information and is involved in decision-making). The CEQ2 is based on a four-point Likert scale ranging from 4 = “totally agree” to 1 = “totally disagree” for 19 questions and on visual analogue scales (VAS) from 0–100 for the last three questions coded according to a range from 1 to 4. The total score is calculated as a mean of all the subscales and ranges from 0 (worse experience) to 4 (more positive experience).

The secondary outcome was PTSD-FC symptoms, assessed using the PCL-5, a 20-item scale based on the Diagnostic and Statistical Manual of Mental Disorders (DSM–5)^15^. Instructions were adapted to refer to the recent childbirth. A validated French version of this scale exists, with a good internal consistency^31^. A total score ≥ 31 is considered as the cut-off for a probable PTSD diagnosis. The PCL-5 score is divided into four subscales: Intrusion (5 items), Avoidance (2 items), Negative alterations in cognition and mood (7 items) and Alterations in arousal or reactivity (6 items), each item ranging from 0 =Not at all to 4 =Extremely. In this study, the Cronbach alpha was calculated as 0.89. Women also completed two questions of the Major Life Events Questionnaire^32,33^ about cases of violence or abuse before birth, as this is a risk factor for PTSD^18^ and for negative childbirth experience^34^.

The third group of outcomes were obstetric outcomes (i.e., gravidity, maternal age at birth, induction of labor, oxytocin augmentation, analgesia, mode of birth) and neonatal outcomes (i. e., Apgar score at 1, 5 and 10 min, birth weight, and neonatal intensive care unit (NICU) admission), all of which were extracted from digital medical records. Data extracted from medical records comprised a medical file number, which was linked in an Excel table to the name of the patient and her RedCap number.

Demographic information (country of origin, civil status, educational background, employment status, and Body Mass Index (BMI)) was collected via self-report questionnaires. Furthermore, this study included all the antenatal classes that women attended, regardless of their specific methods or settings (at the hospital or in private settings) and women self-reported their attendance of antenatal classes and the number of attended sessions.

### Data analysis

Analyses were conducted with SPSS (Statistical Package for Social Sciences, version 26.0). CEQ2, PCL-5, obstetric, and neonatal outcomes were compared between the two groups, participation in antenatal classes (AC) versus no participation in antenatal classes (NAC), using independent sample t tests, Chi2 tests or Fischer exact tests. Bi-variate correlations (One-way ANOVA) were carried out to investigate which variables of interest (including post-hoc exploratory analyses) were related to the childbirth experience. Finally, regression analyses were conducted with all variables that were positively correlated by forcing the variable ‘participation in antenatal classes (yes/no)’ into the regression (stepwise hierarchical regression). For the regression of the CEQ-2 score (dependant variable), the variable ‘participation in antenatal classes’ was entered in the first step as independent variable. In the second step, the other independent variables were entered, including history of violence or abuse in the last two years, civil status, employment status, and time since birth, induction of labor, analgesia, forceps, emergency caesarean, operative delivery (all deliveries except spontaneous vaginal birth), Apgar score at 1 min, Apgar score at 5 min, and NICU admission. For the regression of PCL-5, the same model was used, except for the time since birth, as this was not correlated with the PCL-5 total score.

Depending on the question, the rate of missing responses varied from 16% to 21.7% for the CEQ-2 and from 29 to 29.4% for the PCL-5. Missing data for PCL-5 or CEQ-2 were managed using pairwise deletion at item level. No missing data were replaced. No data were missing for demographic, obstetric or neonatal outcomes.

## Results

Of the 2876 eligible patients contacted, 794 (27.6%) women completed the questionnaires. Non-responders were significantly younger than responders (M = 31.1 years, SD = 5.1 versus M = 32.5 years, SD = 4.4; *p* < 0.001) and differed significantly regarding their employment status, i.e., non-responders were more likely to be unemployed (21.3% versus 5.1%), while responders were more likely to have white-collar jobs (66.9% versus 42.3%). Among the responders, 592 (74.46%) had attended antenatal classes.

Comparing those who had attended antenatal classes (AC) with those who had not (NAC), those in the AC group were older, with a lower weight (*p* < 0.05), more likely to have completed a university education, and to exercise an intellectual and scientific profession (*p* < 0.01; see Table 1).

**Table 1:** Sociodemographic sample characteristics and obstetric and neonatal outcomes (n = 795).

	AC (antenatal classes group) n = 592	NAC (Non antenatal classes group) n = 203	Test result	*p* value
Completion time from birth (days), M ± SD	436.58 ± 216.79	401.56 ± 234.01	1.86^a^	0.52
Maternal age at birth (years), M ± SD	32.9 ± 4.16	31.71 ± 5.02	3.04^a^	0.001
Gravidity , M ± SD	1.31 ± .67	1.40 ± .92	− 1.31^a^	0.13
BMI at birth (weight at birth in kg/ (size in cm)^2^, M ± SD	27.29 ± 9.36	28.31 ± 4.92	− 1.52^a^	0.44
**Country of origin, n (%)**			7.72^b^	0.103
Switzerland	359 (60.6%)	128 (63.1%)		
European Union (UE)	189 (31.9%)	51 (25.1%)		
Europe except UE	13 (2.2%)	10 (4.9%)		
Americas	17 (2.9%)	6 (3%)		
Other countries	14 (2.4%)	8 (3.9%)		
**Civil Status, n (%)**			0.42^b^	0.811
Single/ Separate/Divorced/Widow	182 (31%)	60 (30%)		
Married / In common-law	405 (69%)	140 (70%)		
**Educational background, n (%)**			48.48^b^	0.000
Primary education/Secondary education or other level	9 (1.5%)	18 (9%)		
Apprenticeship	92 (15.7%)	56 (27.9%)		
Higher secondary education	35 (6%)	18 (9%)		
University or higher education	450 (76.8%)	109 (54.2%)		
**Employment status categorized according to Nomenclature suisse des professions CH-ISCO-19, n (%)**			42.65^b^	0.000
Directors, executives managers	28 (4.9%)	13 (6.8%)		
Intellectual and scientific professions	388 (67.8%)	82 (43.2%)		
Intermediate professions	94 (16.4%)	49 (25.8%)		
Administrative type employees	11 (1.9%)	5 (2.6%)		
Staff in direct services to individuals, traders and salespeople	19 (3.3%)	19 (10%)		
Other professions	10 (1.7%)	5 (2.6%)		
No employment	22 (3.8)	17 (8.9%)		
Fertility treatment, n (%)	71 (12%)	21 (10.3%)	0.41^b^	0.522
**Mode of delivery, n (%)**			8.63^b^	0.071
Elective caesarean	35 (5.9%)	22 (10.8%)		
Emergency caesarean	84 (14.2%)	31 (15.3%)		
Forceps	58 (9.8%)	11 (5.4%)		
Vacuum extraction	42 (7.1%)	14 (6.9%)		
Spontaneous birth	373 (63%)	125 (61.6%)		
Induction of labor, n (%)	190 (36.9%)	61 (36.1%)	0.04^b^	0.852
Oxytocin augmentation, n (%)	105 (20.4%)	30 (17.8%)	0.56^b^	0.455
**Analgesia, n (%)**			2.2^b^	0.699
None	36 (6.1%)	11 (5.4%)		
Local anaesthesia, Pudendal Nerve Block, EMONO (Nitrous Oxide/Oxygen 50%/50%)	92 (15.5%)	28 (13.8%)		
Epidural	390 (65.95%)	131 (64.5%)		
Spinal anaesthesia	68 (11.5%)	31 (15.3%)		
General anaesthesia	6 (1%)	2 (1%)		
Apgar at 1 min, M ± SD	8.13 ± 1.95	7.84 ± 2.38	1.57^a^	0.084
Apgar < 7 at 1 min, n (%)	112 (18.9%)	47 (23.1%)	1.69^b^	0.193
Apgar at 5 min, M ± SD	9.38 ± 1.0	9.21 ± 1.32	1.71^a^	0.051
Apgar < 7 at 5 min, n (%)	9 (0.01%)	8 (0.04%)	4.23^c^	0.0496
Apgar at 10 min, M ± SD	9.74 ± .71	9.67 ± .76	1.1^a^	0.258
Birth weight (grams), M ± SD	3305.12 ± 427.87	3283.10 ± 438.25	0.63^a^	0.530

^a^Independent sample t test.

^b^Chi2 test.

^c^Fischer exact test.

With regards to maternal age and migrant status, the study sample was representative of the population in the catchment area of the university hospital (Canton Vaud)^35,36^. The sample over-represented women who had a partner or were married (69.1% in our study versus 49% in the canton of Vaud), as well as women with a high level of education (71% in our study versus 42% in the canton of Vaud^35^.

Across the whole sample, the PCL-5 total score was negatively correlated with the CEQ-2 total score (*p* < 0.001), indicating that a better childbirth experience was associated with a lower risk of PTSD-FC symptoms.

### Childbirth experience

The mean CEQ-2 total score was 3.06 (SD = 0.62). The mean CEQ-2 total score (*p* < 0.05), and the mean CEQ-2 own capacity score (*p* < 0.05) were lower for the AC group compared to the NAC group, which means that women who attended antenatal classes reported a less positive childbirth experience than women who did not attend antenatal classes (see Table 2).

**Table 2:** Results of PCL-5 and CEQ-2 according to antenatal class attendance.

	AC (antenatal classes group) Mean (SD) [Range]	NAC (antenatal classes group) Mean (SD) [Range]	t^a^	*P* value
CEQ-2 total score	3.03 (0.62) [1.1–4]	3.16 (0.57) [1.19–4]	− 2.16	0.031
CEQ-2 Own capacity	2.61 (0.68) [1–4]	2.75 (0.67) [1.13–4]	− 2.22	0.026
CEQ-2 Perceived safety	3.08 (0.75) [1–4]	3.16 (0.71) [1–4]	− 1.3	0.195
CEQ-2 Professional support	3.30 (0.69) [1–4]	3.41 (0.62) [1–4]	− 1.86	0.063
CEQ-2 Participation	3.11 (0.79) [1–4]	3.20 (0.57) [1–4]	− 1.31	0.190
PCL-5 total score	7.50 (8.24) [0–51]	8.68 (9.18) [0–44]	− 1.49	0.138
PCL-5 Intrusion	1.11 (2.08) [0–15]	1.63 (2.84) [0–16]	− 2.10	0.015
PCL-5 Avoidance	0.40 (1.07) [0–8]	0.55 (1.37) [0–7]	− 1.26	0.156
PCL-5 Negative alterations in cognitions and mood	2.78 (3.54) [0–21]	2.80 (3.72) [0–19]	− 0.07	0.945
PCL-5 Alterations in arousal and reactivity	3.18 (3.40) [0–18]	3.79 (4.04) [0–19]	− 1.70	0.065

^a^Independent sample t test.

When calculating bivariate correlations between all study variables, the CEQ-2 total score and CEQ-2 subscale scores Own Capacity, Perceived Safety, and Participation were negatively correlated with: the completion time from birth, induction of labor, forceps emergency caesarean, operative delivery, and low Apgar (< 7) at 1 min. Furthermore, the CEQ-2 total score was positively correlated with civil status. In addition, “Own Capacity” was positively correlated with increasing maternal age at birth. “Own Capacity” and “Perceived Safety” were significantly correlated with type of analgesia: the lower mean score of "Perceived Safety" was associated with rachi-anaesthesia (M = 2.95; SD = 0.72), while the higher mean score of intrusion was associated with local anaesthesia, pudendal nerve block, EMONO (Nitrous Oxide/Oxygen 50%/50%) (M = 3.45; SD = 0.55). No analgesia was associated with a mean score of perceived safety of 3.29 ± 0.66, higher than the overall average score for the whole sample (3.09 ± 0.74). Finally, “Perceived Safety” was also significantly associated with hospitalisation of the newborn in the NICU (see Table 3 for details).

**Table 3:** Bivariate Pearson correlations between the dependent variables (PCL-5, CEQ-2) and sociodemographic, as well as obstetric and neonatal variables.

	CEQ-2 total score	CEQ-2 Own capacity	CEQ-2 Perceived safety	CEQ-2 Professional support	CEQ-2 Participation	PCL-5 total score	PCL-5 Intrusion	PCL-5 Avoidance	PCL-5 Cognitions	PCL-5 Arousal
Maternal age at birth	− 0.05	− 0.1**	− 0.04	0.01	− 0.05	− 0.01	− 0.05	0.01	− 0.00	0.03
BMI at birth	0.02	0.05	− 0.011	0.02	− 0.01	0.04	0.02	0.02	0.04	0.03
Oxytocin augmentation	− 0.02	− 0.02	− 0.00	− 0.01	0.00	0.00	0.01	0.00	0.03	− 0.03
Induction of labor	− 0.20**	− 0.25**	0.18**	− 0.07	− 0.12**	0.09*	0.13**	0.14**	0.05	0.07
Forceps	− 0.14**	− 0.18**	− 0.13**	− 0.03	− 0.08*	0.07	0.12**	0.05	0.06	0.02
Emergency caesarean	− 0.27**	− 0.31**	− 0.28**	− 0.16**	− 0.16**	0.09*	0.17**	0.20**	0.06	− 0.00
Operative delivery	− 0.31**	− 0.33**	− 0.30**	− 0.12**	− 0.13**	0.11	0.19**	0.151**	0.08*	0.03
Analgesia^a^	8.00	14.8**	8.64**	1.97	1.78	2.34	3.51**	2.36	2.02	0.45
Birth weight	0.00	0.01	0.02	0.00	− 0.01	− 0.02	− 0.03	0.05	− 0.00	− 0.05
Low Apgar score < 7 at 1 min	− 0.09*	− 0.12**	− 0.11**	− 0.03	− 0.08*	0.05	0.07	0.08*	0.04	− 0.01
Low Apgar score < 7 at 5 min	− 0.02	− 0.01	− 0.06	0.01	− 0.00	− 0.02	0.04	0.05	− 0.04	− 0.04
Apgar score at 5 min	0.81*	0.07	0.11**	0.02	0.07	− 0.05	− 0.1*	− 0.09*	− 0.03	− 0.01
NICU	− 0.05	− 0.04	− 0.10**	0.00	− 0.02	0.11**	0.14**	0.152**	0.05	0.05
Violence or abuse in the two years preceeding the birth	0.06	0.03	0.04	0.07	0.02	− 0.12**	− 0.06	− 0.03	− 0.14**	− 0.09*
Civil status	0.08*	0.05	0.06	0.09*	0.07	− 0.04	− 0.00	− 0.00	− 0.04	− 0.05
Employment^a^	1.61	1.15	1.57	20.3	1.22	3.39**	2.02	4.01**	3.05**	1.77
Number of sessions	0.04	0.04	0.06	0.01	0.01	− 0.05	− 0.05	− 0.02	0.02	− 0.09
Completion time from birth (j)	− 0.18**	− 0.13**	− 0.14**	− 0.15**	− 0.17**	0.01	0.04	0.05	0.01	− 0.02

^a^One-way ANOVA.

*p < 0.05; **p < 0.0.

A post-hoc analysis of the CEQ-2 scores according to the antenatal classes attendance was done taking into account the data collection time. The mean CEQ-2 total score (*p* < 0.05) and the mean CEQ-2 own capacity score (*p* < 0.05) remained significantly lower in the AC group for a data collection time under 6 months after the birth. However, this difference disappeared for the data collection time up to 6 months after the birth, with the exception of the CEQ-2 subscale “Perceived safety”, which was found to be significantly higher for the NAC group when the data collection time was between 18 to 24 months (supplementary material).

When entering all significant correlations with childbirth experience into a stepwise hierarchical regression, induction of labor, use of forceps, emergency caesarean section, and civil status remained significant negative predictors of childbirth experience, whereas antenatal class attendance was not retained (see Table 4).

**Table 4:** Results of multiple linear regression analyses for variables predicting CEQ-2.

	CEQ-2 total score			CEQ-2 Own capacity			CEQ-2 Perceived safety			CEQ-2 Professional support			CEQ-2 Participation		
	B	β	95% CI	B	β	95% CI	B	β	95% CI	B	β	95% CI	B	β	95% CI
Antenatal classes attendance	− 0.10	− 0.07	− 0.21; 0.02	− 0.11	− 0.07	− 0.23; 0.01	− 0.09	− 0.05	− 0.22; 0.04	− 0.09	− 0.06	− 0.20; 0.03	− 0.07	− 0.04	− 0.21; 0.08
Maternal age at birth				0.00	0.00	− 0.01; 0.01									
Induction of labor	**− 0.15**	**− 0.12**	**− 0.25; − 0.05**	**− 0.21**	**− 0.15**	**− 0.32; − 0.10**	**− 0.13**	**− 0.08**	**− 0.25; − 0.01**				− 0.13	− 0.08	− 0.26; 0.00
Forceps	**− 0.29**	**− 0.14**	**− 0.51; − 0.06**	**− 0.29**	**− 0.13**	**− 0.53; − 0.05**	**− 0.34**	**− 0.14**	**− 0.61; − 0.08**				− 0.19	− 0.07	− 0.48; 0.10
Emergency caesarean	**− 0.48**	**− 0.24**	**− 0.69; − 0.26**	**− 0.51**	**− 0.23**	**− 0.74; − 0.27**	**− 0.56**	**− 0.25**	**− 0.82; − 0.31**	**− 0.27**	**− 0.14**	**− 0.44; − 0.11**	− 0.25	− 0.11	− 0.53; 0.02
Operative delivery	− 0.02	− 0.02	− 0.19; 0.15	− 0.18	− 0.12	− 0.36; 0.01	− 0.08	− 0.05	− 0.28; 0.13	− 0.05	− 0.04	− 0.17; 0.07	− 0.03	− 0.02	− 0.25; 0.19
Analgesia	0.01	0.03	− 0.01; 0.03	0.02	0.07	− 0.00; 0.05	0.02	0.05	− 0.01; 0.05						
Low Apgar score < 7 at 1 min	− 0.03	− 0.02	− 0.17; 0.12	− 0.07	− 0.04	− 0.20; 0.07	0.01	0.01	− 0.16; 0.18				− 0.09	− 0.05	− 0.25; 0.06
Apgar score at 5 min	0.03	0.04	− 0.03; 0.08				0.03	0.05	− 0.03; 0.10						
NICU							− 0.13	− 0.05	− 0.32; 0.06						
Violence or abuse in the two years preceding the birth															
Civil status	**0.12**	**0.09**	**0.02; 0.22**							**0.14**	**0.09**	**0.03; 0.24**			
Employment															
Completion time from birth	0.00	− 0.16	− 0.00; 0.00	0.00	− 0.10	− 0.00; 0.00	0.00	− 0.11	− 0.00; 0.00	0.00	− 0.14	− 0.01; 0.000	0.00	− 0.14	− 0.00; 0.00
R^2^	0.16			0.21			0.15			0.06			0.04		

B = unstandardized regression coefficient; β = standardized regression coefficient; 95% CI = 95% bias corrected and accelerated confidence intervals of unstandardized regression coefficient as estimated by means of bootstrapping. Bold regression coefficients are significantly different from 0 (*p* < .05). CEQ-2 = Childbirth Experience Questionnaire 2.

### PTSD-FC

The mean PCL-5 total score was 7.89 (SD = 0.58). Twenty (3.26%) participants had a total score ≥ 31, which represents the cut-off for a probable PTSD diagnosis. The mean Intrusion score was lower in the AC group (*p* < 0.05) compared to the NAC group (see Table 2). The total score and subscale scores Intrusion and Avoidance were positively correlated with an induction of labor, an emergency caesarean, and NICU admission. In addition, PCL-5 total score and subscale scores Avoidance and Cognitions were positively correlated with employment status. The PCL-5 total score and subscale scores Cognitions and Arousal were negatively correlated with violence or abuse in the last two years before birth. Intrusion, Avoidance, and Cognitions were positively correlated with operative delivery. Intrusion was correlated with analgesia: the higher mean score of intrusion was associated with general anaesthesia (M = 1.83;SD = 1.94), while the lower mean score of intrusion was associated with local anaesthesia, pudendal nerve block, EMONO (Nitrous Oxide/Oxygen 50%/50%) (0.55 ± 1.64). No analgesia during childbirth was associated to a mean score of intrusion (M = 1.19; SD = 2.11), lower than the mean score for the whole sample (M = 1.28; SD = 2.34); Avoidance was positively correlated with low Apgar (< 7) at 1 min, while Intrusion and Avoidance were negatively correlated with Apgar score at 5 min (see Table 3).

As the influence of time on the PCL-5 score was already known, a post-hoc analysis of the scores according to the antenatal classes' attendance was done, taking into account the data collection time. PCL-5 Intrusion and Avoidance scores were significantly lower in the AC group for a data collection time under 6 months after the birth. Six to 12 months after the birth, all the PCL-5 scores were significantly lower in the AC group. After 12 months, only the PCL-5 Avoidance score was lower in the AC group when the data collection time was from 18 to 24 months after the birth but was higher for the AC group when the data collection time was from 12 to 18 months and after 24 months after the birth (supplementary material).

When entering all significant correlations with PCL-5 into multiple linear regressions (stepwise hierarchical regression), antenatal class attendance remained a significant predictor of the PCL-5 Intrusion score, as well as the use of forceps, emergency caesarean, and NICU admission (see Table 5).

**Table 5:** Results of multiple linear regression analyses for variables predicting PCL-5.

	PCL-5 total score			PCL-5 Intrusion			PCL-5 Avoidance			PCL-5 Cognitions			PCL-5 Arousal		
	B	β	95% CI	B	β	95% CI	B	β	95% CI	B	β	95% CI	B	β	95% CI
Antenatal classes attendance	− 1.35	− 0.07	− 3.31; 0.61	**− 0.46**	**− 0.09**	**− 0.92; − 0.01**	− 0.02	− 0.01	− 0.28; 0.23	− 0.02	− 0.01	− 0.24; 0.20			
Induction of labor	1.44	0.08	− 0.27; 3.15	0.28	0.06	− 0.14; 0.70	**0.23**	**0.10**	**0.00; 0.45**						
Forceps				**0.93**	**0.12**	**0.00; 1.85**									
Emergency caesarean	1.41	0.05	− 1.62; 4.44	**0.91**	**0.13**	**0.03; 1.78**	**0.47**	**0.13**	**0.07; 0.86**						
Operative delivery	1.45	0.08	− 0.64; 3.54	0.33	0.06	− 0.38:1.04	0.22	0.09	− 0.56; 0.50	**0.37**	**0.16**	**0.19; 0.57**			
Analgesia				− 0.08	− 0.07	− 0.17; 0.02									
Low Apgar score < 7 at 1 min							− 0.08	− 0.03	− 0.40; 0.24						
Apgar score at 5 min				− 0.05	− 0.02	− 0.24; 0.14	− 0.04	− 0.04	− 0.16; 0.08						
NICU	1.36	0.05	− 1.41; 4.14	**0.85**	**0.11**	**0.17; 1.52**	0.30	0.07	− 0.08; 0.67						
Violence or abuse in the two years preceding the birth	**− 2.21**	**− 0.12**	**− 3.96; − 0.47**				− 0.1	− 0.04	− 0.32; 0.16	− 0.07	− 0.03	− 0.26; 0.12			
Civil status															
Employment							**0.10**	**0.16**	**0.04; 0.16**	**0.10**	**0.16**	**0.05; 0.15**			
R^2^	0.08			0.09			0.1			0.05					

B = unstandardized regression coefficient; β = standardized regression coefficient; 95% CI = 95% bias corrected and accelerated confidence intervals of unstandardized regression coefficient as estimated by means of bootstrapping. Bold regression coefficients are significantly different from 0 (*p* < .05). PCL-5 = Posttraumatic Stress Disorder Checklist for DSM 5.

### Obstetric and neonatal outcomes

No significant differences were found between both groups (AC and NAC) regarding obstetric outcomes (fertility treatment, mode of delivery induction of labor, oxytocin augmentation) or neonatal outcomes (Apgar scores and the birth weight of the baby). Less newborns with low Apgar scores (< 7) at 5 min were reported in the AC group, with a trend towards significance (*p* = 0.05). Regarding especially the use of analgesia, no significant difference was found between the AC and NAC groups (see Table 1).

## Discussion

This cross-sectional study compared women regarding their childbirth experience, their PTSD-FC symptoms, as well as their obstetrical and neonatal outcomes according to their participation or not in antenatal classes (AC vs. NAC). Women who attended antenatal classes had a poorer childbirth experience but were less likely to develop birth-related intrusion symptoms. Obstetric and neonatal outcomes were comparable between both groups.

Women who had attended antenatal classes reported a more negative childbirth experience, even though most of the factors associated with the childbirth experience (frequency of induction of labor, use of forceps, and emergency cesarean section) did not differ between those two groups. Studies so far showed inconsistent results regarding the link between antenatal class attendance and childbirth experience^34,37,38^. There may be different explanations for this. First, data collection occurred at different time points in different studies, from five months to five years after the childbirth, and comparisons between studies are therefore difficult. Little is actually known about the role that time since childbirth plays in the reporting of the childbirth experience. Using a five-point Likert scale, Maimburg and colleagues, showed that only 51% of women gave the same evaluation of their childbirth experience over time (from six weeks post-partum to five years post-partum); for 40% of them, the score decreased over time, while for 9% of participants, the childbirth experience score increased over time^39^. In our study, the data collection occurred from 45 days to two years after chilbirth. This variability regarding time since childbirth may have influenced our results, as we find a negative corelation between CEQ-2 total score and subscales and completion time since birth. The post-hoc analysis of the CEQ-2 scores according to the antenatal class attendance, taking into account the data collection time, shows the importance of the time that passes between the birth and the completion of the questionnaire, since the only significant difference in the birth experience scores was seen for women who completed the questionnaire less than 6 months after the birth. Second, differences in the content of the antenatal classes could contribute to explaining those differences in childbirth experience across the studies. For example, depending on the content of the antenatal classes, mothers’ sense of control may be strengthened^34^. However, if the content of the antenatal classes is not matched with the reality of birth, mothers may develop unrealistic expectations^34^; the non-fulfilment of these expectations may in turn negatively affect their childbirth experience^40^.

The prevalence of 3.26% of patients with PTSD-FC symptoms in our sample is in line with prevalence rates found in community samples^13,14^. Compared to women who did not attend antenatal classes, women who attended antenatal classes had less symptoms of intrusion. The post-hoc analysis shows that for women who gave birth less than 6 months before completing the survey, the intrusion and avoidance scores are significantly lower in the AC group as all PCL-5 scores for women who gave birth between 6 and 12 months before completing the survey. The PCL-5 score is the highest of the study in the NAC group for women who completed the questionnaire between 6 and 12 months after the birth (M = 10.73 (± 11.44)). One possible explanation is that the childbirth occurred during the COVID-19 period. However, only 18.3% of the births in this sub-sample took place during the semi-lockdown period (16 March 2020 to 19 June 2020) and the PCL-5 score for the antenatal classes group is the lowest of the study (M = 7.06 ± 7.46). We therefore can only conclude that the relation between PTSD-FC symptoms and antenatal class attendance is more marked between 6 and 12 months after the birth. It is also important to note that PCL-5 scores are relatively higher the more time had passed since the birth in both groups (with the exception of the NAC group between 6 and 12 months after birth). The literature suggests that PCL-5 scores tend to decline over time^41^. The fact that our results show a different trend leads to the question of a possible selection bias.Women in the NAC group in our study reported a significantly lower educational level, a factor also known to predispose them to a higher risk of PTSD-FC^42^. In Switzerland, access to antenatal classes is not free of charge, which may have excluded women with lower financial means. Moreover, women in the AC group were older than those in the NAC group, which may have increased their risk of PTSD-FC^13^. However, we found higher PCL-5 scores (total and subscales) in the younger group (NAC) and no correlation between age and PCL-5 score. The link between birth preparation and PTSD-FC symptoms therefore appears to be very complex to measure, as many individual variables are involved.

No differences were found for obstetric or neonatal outcomes between the AC and the NAC groups, except for APGAR scores at 5 min of life, which were better in the AC group. The overall rate of cesarean sections in the study sample was 21.6%. This is slightly lower than the hospital rate of 27% during the study period^43^, which comprises term and preterm, primiparous, and multiparous cesarean sections. Other studies have reported conflicting results about the associations between antenatal class attendance and the mode of delivery^44^. This implies again that the format and content of the antenatal classes is important. Yet, it seems that the most optimal format has not yet been found and scientifically validated^44^. Regarding the use of analgesia and particularly the use of an epidural, this study showed no significant differences between groups. Even if the main historical outcome of the antenatal classes was the management of pain, in this study, as well as in others, no significant associations between birth preparedness and reported pain level during labor or the use of analgesics during labor were found^45–47^.

One of the strengths of this study is that it did not seek to evaluate a specific birth preparation program but investigated associations between antenatal classes whatever the type of antenatal classes and childbirth experience, PTSD-FC symptoms, obstetrical, and neonatal outcomes in a routine clinical context. Another strength of our study was the use of multiple validated questionnaires in French and English in order to access a larger population than only the French-speaking population. The large sample size is higher than the a-priori sample size calculation, which reinforces the power of the analysis.

However, this study has a number of limitations. First, it is a retrospective study, and a retrospective bias to the reporting of outcomes may therefore not be excluded. Second, given the cross-sectional desing, no causal relationships could be investigated. Moreover, this design did not allow us to take into account an important variable: the emotional state of the women before the birth, including any previous traumatic experience or PTSD. Furthermore, this study included all the antenatal classes that women attended, regardless of their specific methods or settings (at the hospital or in private settings) and women self-reported their attendance in antenatal classes. Additionally, the low response rate (27.6%) is also an important limitation, although it is comparable to other questionnaire studies^20,48,49^, particularly to online surveys^50,51^. It is possible that women with traumatic childbirth experiences, particularly following emergency caesarean sections, did not participate in this study. Of note, preterm deliveries, known to increase the risk of PTSD, were excluded from this study. Finally, the fact that the questionnaire was only proposed in two languages (French and English) excluded women who did not have sufficient mastery of those languages.

Future studies should prospectively investigate the effect of specific elements of antenatal classes (format, content, setting, etc.) on the risk of PTSD-FC, ideally employing a randomised controlled trial design.

## Conclusion

This study shows that women who attended antenatal classes had a poorer childbirth experience but were less likely to develop birth-related intrusion symptoms. Obstetric or neonatal outcomes were similar between both groups. The result related to the childbirth experience raises questions about the match between the content of the sessions, the reality of the needs and expectations of women, and the possibilities offered by the birth centres. It seems necessary to define which methods, contents, and tools would promote a better childbirth experience.

## Supplementary Information

Supplementary Information.

## Data Availability

The datasets used and/or analysed during the current study available from the corresponding author on reasonable request.
