# Author Correction: Childbirth experience, risk of PTSD and obstetric and neonatal outcomes according to antenatal classes attendance

**DOI:** 10.1038/s41598-022-16464-0

**Published:** 2022-07-13

**Authors:** Valérie Avignon, David Baud, Laurent Gaucher, Corinne Dupont, Antje Horsch

**Affiliations:** 1 Department Woman-Mother-Child, Lausanne University Hospital (CHUV), Avenue Pierre-Decker 2, 1011 Lausanne, Switzerland; 2 Research On Healthcare Performance RESHAPE, INSERM U1290, Université Claude Bernard Lyon 1, Domaine Rockefeller 8 avenue Rockefeller 69373 Lyon cedex 08, Lyon, France; 3 Institute of Higher Education and Research in Healthcare (IUFRS), University of Lausanne, Route de la Corniche 10, 1010 Lausanne, Switzerland; 4 Geneva School of Health Sciences, HES-SO University of Applied Sciences and Arts, Western Switzerland, Geneva, Switzerland

Correction to: *Scientific Reports* 10.1038/s41598-022-14508-z, published online 23 June 2022

The original version of this Article contained an error in the spelling of the authors Valérie Avignon, David Baud, Laurent Gaucher, Corinne Dupont & Antje Horsch, which were incorrectly given as Avignon Valérie, Baud David, Gaucher Laurent, Dupont Corinne & Horsch Antje.

The original Article has been corrected.

