# Supplementary material for: Childbirth experience, risk of PTSD and obstetric and neonatal outcomes according to antenatal classes attendance
Source: Sci Rep. 2022 Jun 23;12:10717. doi: 10.1038/s41598-022-14508-z (PMC9225805; doi:10.1038/s41598-022-14508-z)
Supplement: Supplementary file 1 — Supplementary Information. [file 41598_2022_14508_MOESM1_ESM.pdf]

Childbirth experience, risk of PTSD and obstetric and neonatal outcomes according to antenatal classes attendance

Avignon Valérie[1,2,3*], Baud David[1], Gaucher Laurent[2,4], Dupont Corinne[2], Horsch Antje [1,3]

1. Department Woman-Mother-Child, Lausanne University Hospital (CHUV), Avenue Pierre-Decker 2, 1011, Lausanne, Switzerland.
2. Research on Healthcare Performance RESHAPE, INSERM U1290, Université Claude Bernard Lyon 1, France.
3. Institute of Higher Education and Research in Healthcare (IUFRS), University of Lausanne, Route de la Corniche 10, 1010, Lausanne, Switzerland
4. Geneva School of Health Sciences, HES-SO University of Applied Sciences and Arts, Western Switzerland, Geneva, Switzerland.

[*]Corresponding author

Supplementary material: Results of PCL-5 and CEQ-2 according to antenatal class attendance and completion time from birth

Completion time from birth <6 months	AC (antenatal classes group) Mean (SD) n = 63	NAC (antenatal classes group) Mean (SD) n = 46	t[1]	*P value*
**CEQ-2 total score**	**3.22 (0.52)**	**3.32 (0.53)**	**-2.16**	**0.031**
**CEQ-2 own capacity**	**2.74 (0.65)**	**2.89 (0.70)**	**-2.22**	**0.026**
CEQ-2 perceived safety	3.28 (0.59)	3.36 (0.70)	-1.3	0.195
CEQ-2 professional support	3.48 (0.61)	3.59 (0.48)	-1.86	0.063
CEQ-2 participation	3.35 (0.60)	3.50 (0.63)	-1.31	0.190
PCL-5 total score	7.71 (7.58)	7.89 (7.62)	-0.12	0.802
**PCL-5 intrusion**	**0.79 (1.47)**	**1.65 (3.29)**	**-1.84**	**0.000**
**PCL-5 avoidance**	**0.13 (0.52)**	**0.30 (0.99)**	**-1.21**	**0.015**
PCL-5 Negative alterations in cognitions and mood	2.95 (3.52)	2.35 (3.14)	0.93	0.707
PCL-5 Alterations in arousal and reactivity	3.94 (3.42)	3.59 (3.62)	0.52	0.730
Completion time from birth from 6 to 12 months	AC Mean (SD) n =154	NAC Mean (SD) n = 48	t[1]	*P value*
CEQ-2 total score	3.11 (0.58)	3.09 (0.59)	0.26	0.947
CEQ-2 own capacity	2.69 (0.68)	2.68 (0.68)	0.02	0.652
CEQ-2 perceived safety	3.15 (0.72)	3.07 (0.77)	0.65	0.718
CEQ-2 professional support	3.37 (0.65)	3.30 (0.65)	0.68	0.937
CEQ-2 participation	3.17 (0.77)	3.05 (0.78)	0.93	0.806
**PCL-5 total score**	**7.06 (7.46)**	**10.73 (11.44)**	**-2.60**	**0.000**
**PCL-5 intrusion**	**0.94 (1.67)**	**2.06 (2.94)**	**-3.38**	**0.000**
**PCL-5 avoidance**	**0.39 (1.07)**	**0.96 (1.83)**	**-2.70**	**0.000**
**PCL-5 Negative alterations in cognitions and mood**	**2.75 (3.50)**	**3.36 (4.44)**	**-1.00**	**0.038**
**PCL-5 Alterations in arousal and reactivity**	**2.92 (3.13)**	**4.17 (4.46)**	**-2.16**	**0.003**
Completion time from birth from 12 to 18 months	AC Mean (SD) n = 87	NAC Mean (SD) n = 14	t[1]	*P value*
CEQ-2 total score	3.03 (0.63)	3.08 (0.62)	-0.4	0.860
CEQ-2 own capacity	2.58 (0.69)	2.84 (0.61)	-1.56	0.327
CEQ-2 perceived safety	3.04 (0.77)	3.12 (0.71)	-0.41	0.840
CEQ-2 professional support	3.34 (0.63)	3.34 (0.67)	-0.02	0.527
CEQ-2 participation	3.17 (0.77)	3.10 (0.94)	0.41	0.206
PCL-5 total score	7.43 (8.29)	5.64 (5.24)	0.78	0.135
PCL-5 intrusion	1.19 (2.33)	1.31 (2.50)	-0.19	0.870
**PCL-5 avoidance**	**0.58 (1.38)**	**0.00 (0.00)**	**1.63**	**0.002**
PCL-5 Negative alterations in cognitions and mood	2.56 (3.42)	2.21 (2.26)	0.37	0.309
PCL-5 Alterations in arousal and reactivity	2.98 (2.99)	3.19 (3.51)	-0.25	0.733
Completion time from birth from 18 to 24 months	AC Mean (SD) n = 90	NAC Mean (SD) n = 35	t[1]	*P value*
CEQ-2 total score	2.92 (0.63)	3.06 (0.51)	-1.20	0.228
CEQ-2 own capacity	2.50 (0.67)	2.66 (0.60)	-1.29	0.026
**CEQ-2 perceived safety**	**3.02 (0.72)**	**3.12 (0.52)**	**-0.74**	**0.017**
CEQ-2 professional support	3.17 (0.76)	3.41 (0.62)	-0.96	0.476
CEQ-2 participation	2.94 (0.80)	3.09 (0.73)	-1.08	0.471
PCL-5 total score	7.60 (8.69)	7.91 (8.73)	-0.18	0.700
PCL-5 intrusion	1.37 (2.28)	1.37 (2.49)	-0.10	0.521
**PCL-5 avoidance**	**0.31 (0.86)**	**0.63 (1.42)**	**-1.53**	**0.005**
PCL-5 Negative alterations in cognitions and mood	2.84 (3.62)	2.43 (3.74)	0.57	0.495
PCL-5 Alterations in arousal and reactivity	3.08 (3.59)	3.49 (3.75)	-0.56	0.994
Completion time from birth up to 24 months	AC Mean (SD) n = 52	NAC Mean (SD) n = 11	t[1]	*P value*
CEQ-2 total score	2.87 (0.72)	3.19 (0.64)	-1.64	0.259
CEQ-2 own capacity	2.54 (0.79)	2.61 (0.81)	-0.35	0.994
CEQ-2 perceived safety	2.91 (0.89)	3.10 (0.89)	-0.80	0.923
CEQ-2 professional support	3.13 (0.75)	3.64 (0.62)	-2.55	0.170
CEQ-2 participation	2.90 (0.92)	3.20 (0.72)	-1.29	0.138
PCL-5 total score	8.52 (10.3)	9.36 (8.96)	-0.25	0.871
PCL-5 intrusion	1.43 (2.83)	0.92 (1.89)	0.61	0.536
**PCL-5 avoidance**	**0.55 (1.22)**	**0.23 (0.44)**	**0.92**	**0.048**
PCL-5 Negative alterations in cognitions and mood	2.91 (3.81)	4.09 (3.72)	-0.94	0.964
PCL-5 Alterations in arousal and reactivity	3.54 (4.31)	4.69 (5.39)	-0.82	0.292
